# Social threat indirectly increases moral condemnation via thwarting fundamental social needs

**DOI:** 10.1038/s41598-021-00752-2

**Published:** 2021-11-05

**Authors:** Robert K. Henderson, Simone Schnall

**Affiliations:** Department of Psychology, University of Cambridge, Cambridge, UK

**Keywords:** Psychology, Human behaviour

## Abstract

Individuals who experience threats to their social needs may attempt to avert further harm by condemning wrongdoers more severely. Three pre-registered studies tested whether threatened social esteem is associated with increased moral condemnation. In Study 1 (*N* = 381) participants played a game in which they were socially included or excluded and then evaluated the actions of moral wrongdoers. We observed an indirect effect: Exclusion increased social needs-threat, which in turn increased moral condemnation. Study 2 (*N* = 428) was a direct replication, and also showed this indirect effect. Both studies demonstrated the effect across five moral foundations, and was most pronounced for harm violations. Study 3 (*N* = 102) examined dispositional concerns about social needs threat, namely social anxiety, and showed a positive correlation between this trait and moral judgments. Overall, results suggest threatened social standing is linked to moral condemnation, presumably because moral wrongdoers pose a further threat when one’s ability to cope is already compromised.

Loss of social connections is a perpetual risk when interacting with others. Indeed, it has been suggested that people possess an internal ‘sociometer’ that tracks self-esteem relating to one’s social position amongst peers^1,2^. Threats to self-esteem are associated with feeling foolish, awkward, and inadequate, indicating that self-esteem may be a proxy for the appreciation that individuals believe others feel towards them. Further evidence suggests that the sociometer monitors the social environment for cues indicating social acceptance, and, when such cues are absent, individuals subsequently experience negative affect, such as social anxiety^2,3^. Sensitivity to social disapproval exists even in brief interactions with strangers that one will never meet face-to-face^2,4,5^. If individuals experience threats to their social needs—namely, social threat—then these adaptive mechanisms monitor the environment for information relevant to restoring such needs^6^.

In fact, Dickerson and Kemeny^7^ proposed a ‘social self-preservation system’ that continually scans the environment for potential threats to social standing. In a meta-analysis of 208 studies on cortisol responses to stressful situations they found that tasks with the potential for negative social evaluation (e.g., delivering a speech) resulted in cortisol levels more than three times higher than non-social stressful tasks (e.g., mental arithmetic). They conclude that individuals may be equally concerned with preserving their social needs as their physical safety, because cortisol rises to recruit physiological energy for a fight or flight response in the face both social and physical threats.

We propose that thwarted social needs—that is, heightened social threat—fortifies moral judgments across moral domains, rather than a single domain (e.g., fairness). This is consistent with error management, such that if an individual is in a heightened state of danger as a consequence of social exclusion, the more costly error is to be under-vigilant about some sinister acts than to be over-vigilant for others^8^. That is, individuals who experience social threat may condemn wrongdoers more harshly regardless of moral domain, because underestimating the potential threat from a transgressor of any kind could be particularly costly when one’s social needs have been undermined. In line with the proposed social monitoring and social self-preservation systems, research has shown that social exclusion (vs. inclusion) is associated with bolstered memory for social information^9^**,** greater attention to others’ vocal tones^10^, improved ability to distinguish authentic from inauthentic emotions^11^ and increased aptitude for detecting deception^12^. Thus, social threat can enhance the capacity to identify further sources of potential threat—especially in social contexts.

Furthermore, socially threatened individuals shift towards a “prevention focus,” in which they aim to minimize dangers and risks, as opposed a “promotion focus,” aimed at taking risks to cultivate positive outcomes^13^. That is, individuals who reported experiencing chronic loneliness and participants who experienced social exclusion in lab settings were more cautious, relative to those who reported greater social connection. Furthermore, a meta-analysis indicated that individuals who were more concerned with safety and security relative to growth and advancement made stronger affect-based, deontological judgments in response to moral dilemmas^14^. Conversely, individuals who report stronger social bonds subsequently made less affect-laden, more utilitarian moral judgments^15^. Relatedly, the neural substrates involved in utilitarian moral judgments appear to overlap with brain regions associated with risky decision-making^16^. Taken together, these findings suggest that when people feel safe, they may be willing to take on more risk. In contrast, when individuals experience threat, they might then become more cautious. Based on this existing research, we therefore propose that social threat should be linked to moral condemnation, such that relative to feeling socially secure, experiencing social threat will give rise to harsher judgments of moral wrongdoing.

## Study 1

The first pre-registered study used the classic Cyberball procedure^4,5^ to examine whether social threat increases moral disapproval. Participants rated their disapproval of moral violations, drawn from Moral Foundations Theory^17^, which proposes that morality is an “interlocking set of values, practices, institutions, and evolved psychological mechanisms that work together to suppress or regulate selfishness and make social life possible” and posits at least five moral foundations: Dislike for suffering of others (Harm), concern with cheating and lack of reciprocity (Fairness), group adherence (Loyalty), deference to leadership and tradition (Authority), and concern with purity and contamination (Sanctity). These five foundations are thought to have arisen to cope with adaptive challenges in human ancestral environments. Based on results from a pilot study (see supplement), we predicted stronger effects for harm violations. All studies were approved by the University of Cambridge’s Department of Psychology Ethics Committee and participants in each study gave their informed consent beforehand. All studies were performed in accordance with relevant guidelines and regulations.

### Methods

#### Participants

Participants were recruited via Amazon’s Mechanical Turk. Participants were required to have completed at least 100 tasks, hold a task approval rating of at least 95%, and reside in the U.S. Informed by results from a pilot study in which we detected a suggestive effect of *d* = 0.24 for harm violations, a G*Power analysis^18^ indicated a sample size of 432 for a between-subjects design with 80% power at α = 0.05 to obtain such an effect. Data were collected from 445 participants in anticipation of some participants not complying with instructions. We excluded eight for failing to provide consent prior to the start of the task, and fifty-six for incorrect responses to both of the two manipulation check items. No other participants were excluded. The final sample comprised 381 participants (169 women; age: *M* = 37.11 years, *SD* = 11.77). The inclusion condition had 80 women and 101 men (age: *M* = 37.51 years, *SD* = 12.20) and the exclusion condition had 89 women and 111 men (age: *M* = 36.75 years, *SD* = 11.39).

#### Procedure

Participants were randomly assigned to either the inclusion or exclusion condition in the Cyberball procedure^4,5^, which was introduced as a computer game involving a mental visualization task. Participants controlled a cartoon character and took turns throwing a ball with two other “players” (digital confederates). They were instructed to mentally visualize the game and imagine they were playing the game “in real life.” In the inclusion condition the two other “players” frequently threw the ball to the participant whereas in the exclusion condition they almost exclusively passed the ball only between themselves. Next, participants responded to 60 Moral Foundations Vignettes that had been pre-tested and standardized^19^. There were 12 violations for each foundation, rated on a scale from 1 (not at all wrong) to 5 (extremely wrong). Scenarios included, “You see a girl laughing when she realizes her friend’s dad is the janitor” (Harm), “You see a tenant bribing a landlord to be the first to get their apartment repainted” (Fairness), “You see a man leaving his family business to go work for their main competitor” (Loyalty), “You see a star player ignoring her coach's order to come to the bench during a game” (Authority), and “You see a girl and her sister making out with each other just for practice.” (Sanctity). Vignettes were administered in a randomized order.

Though not pre-registered in this study, as an exploratory measure participants then reported their level of fundamental social needs using a well-established measure commonly used with the Cyberball paradigm^4^ that covers feelings of belonging, self-esteem, meaningful existence and sense of control experienced during the Cyberball game. There were 20 items in total, rated on a scale from 1 (not at all) to 9 (very much so). Items included “I felt rejected” (belonging), “I felt insecure” (self-esteem), “I felt invisible” (meaningful existence), and “I felt the other players decided everything” (sense of control). As a manipulation check, participants estimated how many times they received the ball, and whether they were included as much as the other players in the game. On an exploratory basis they then completed a brief personality inventory to test whether Big Five personality traits may serve as moderators (see analyses in the online supplement). Then they were debriefed and compensated.

The pre-registration is here: http://aspredicted.org/blind.php?x=pu9xy2. We report all measures, manipulations and exclusions.

### Results

#### Confirmatory analyses

##### Manipulation check

As expected, socially included participants reported a higher percentage of throws they received (*M* = 37%, 95% CI = [36%, 38%]) than excluded participants (*M* = 15%, 95% CI = [13%, 16%]), *t*(379) =  − 26.69, *p* < 0.001. Additionally, they accurately reported more inclusion in the game (*M* = 5.21, 95% CI = [4.88, 5.55]) than excluded participants (*M* = 2.23, 95% CI = [1.89, 2.56]), *t*(379) =  − 12.52, *p* < 0.001. Thus, the manipulation was effective.

##### Social Needs-threat

To test the effect of the manipulation, we measured differences in the four fundamental needs between the included and excluded participants. Nine items were reverse scored before all items were averaged into a composite “social needs” score. Cronbach’s alpha across all four needs was 0.93 (0.89 for belonging, 0.84 for self-esteem, 0.90 for meaningful existence, and 0.71 for sense of control), indicating a high level of internal consistency. As predicted, excluded participants reported lower social needs (*M* = 3.01, 95% CI = [2.81, 3.22]) than included participants (*M* = 5.66, 95% CI = [5.44, 5.88]), *t*(379) =  − 17.44, *p* < 0.001, confirming that playing the game yielded the desired effect.

##### Moral judgment

Normality tests revealed that one sanctity item fit the criteria for substantial skewness^20^, and as planned in the pre-registration, was excluded from analyses. For each participant, the average moral disapproval rating across the five foundations was calculated, with higher scores indicating more severe condemnation.

A two-way ANOVA with moral foundation (Harm, Fairness, Authority, Loyalty, and Sanctity) as a within-subjects factor and condition (Inclusion vs. Exclusion) as a between-subjects factor showed a main effect of moral foundation, *F*(1, 379) = 79.81, *p* < 0.001, η^2^ = 0.17, with the highest condemnation for sanctity (*M* = 3.55, 95% CI = [3.48, 3.62]), followed by fairness (*M* = 3.43, 95% CI = [3.36, 3.49]), harm (*M* = 3.27, 95% CI = [3.20, 3.634]), authority (*M* = 3.21, 95% CI = [3.14, 3.28]), and loyalty (*M* = 3.06, 95% CI = [2.98, 3.14]). Contrary to prediction, however, the conditions did not differ, *F*(1, 379) = 0.02, *p* = 0.895, η^2^ = 0.00, and there was no interaction between vignette type and condition, F(1, 379) = 0.542, *p* = 0.462, η^2^ = 0.00. Thus, social exclusion on its own did not directly increase moral condemnation.

#### Exploratory analyses

##### Social needs-threat as a possible mediator

As an exploratory analysis, we used bootstrapping mediation analyses^21^ to test whether exclusion had an indirect effect on moral judgment via thwarted social needs. Indeed, there is an increasing consensus that a total effect of an independent variable on a dependent variable is not a necessary prerequisite for searching for evidence of indirect effects, because theoretically meaningful indirect effects can emerge even in the absence of statistically significant total effects^21–24^.

**Figure 1:**
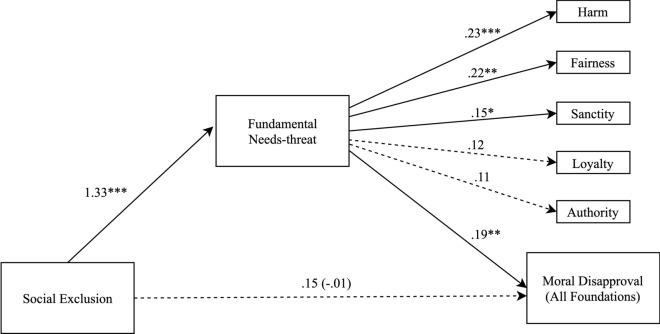
Indirect effect of social threat through fundamental needs-threat on moral disapproval for each moral foundation and across all moral violations in Study 1. Note *p < .05, **p < .01, ***p < .001. Solid lines are significant paths. Values are standardized path coefficients.

Conditional on the assumption that exclusion increases needs-threat, and needs-threat increases moral disapproval, bootstrapping with 5,000 samples suggested that needs-threat mediated the relationship between exclusion and moral disapproval (*b* = 0.16, 95% CI = [0.04, 0.27]). The confidence interval excluded zero, indicating that the indirect effect was significant. Figure 1 shows standardized path coefficients and Table 1 reports the direct and indirect effects linking moral disapproval, needs-threat, and exclusion.

**Table 1:** Study 1 Regression results for the proposed mediation of the effect of exclusion on moral disapproval by needs-threat.

Model	Estimate	SE	*p*	CI (lower)	CI (upper)
**Model without proposed mediator**					
Intercept	3.30	.05	< .001	3.21	3.39
Exclusion → Moral Disapproval (*c*)	−.01	.06	.895	−.13	.11
R^2^_Y,X_	.00	–	–	–	–
**Model with proposed mediator**					
Intercept	3.63	.13	< .001	3.39	3.88
Exclusion → Needs-threat (*a*)	2.65	.15	< .001	2.35	2.95
Needs-threat → Moral Disapproval (*b*)	.06	.02	.005	.02	.10
Exclusion → Moral Disapproval (*c*′)	.15	.08	.074	−.01	.31
Indirect effect (*a* X *b*)	.16	.06	–	.04	.27
Standardized indirect effect (*a* X *b*)	.26	.10	–	.07	.45
R^2^_M,X_	.45	–	–	–	–
R^2^_Y,MX_	.02	–	–	–	–

Regression weights a, b, c, and c′ are illustrated in Fig. 1. R^2^_Y,X_ is the proportion of variance in Y explained by X, R^2^_M,X_ is the proportion of variance in M explained by X, and R^2^_Y,MX_ is the proportion of variance in Y explained by X and M. The 95% CI for *a* X *b* is obtained by the bias-corrected bootstrap with 5,000 resamples. Exclusion is the independent variable (X), Needs-threat is the proposed mediator (M), and Moral Disapproval is the outcome (Y). CI (lower = lower bound of 95% confidence interval; CI (upper) = upper bound.

##### Moral foundations and needs-threat

Further bootstrapping analyses examined the indirect effect of exclusion on individual moral foundations via needs-threat. On the assumption of our causal model that exclusion increases needs-threat, which increases moral disapproval, there was a significant indirect effect for harm (*b* = 0.21, *SE* = 0.07; 95% CI = 0.08 to 0.34), fairness (*b* = 0.19, SE = 0.06; 95% CI = 0.07 to 0.31), and sanctity (*b* = 0.15, SE = 0.07; 95% CI = 0.01 to 0.28). In contrast, although in the same direction, there were no significant effects for authority (*b* = 0.10, SE = 0.07; 95% CI =  − 0.03 to 0.23) or loyalty (*b* = 0.13, SE = 0.08; 95% CI =  − 0.02 to 0.28).

### Discussion

Although there was no main effect of social threat on moral judgment, an exploratory analysis revealed an indirect effect based on our suggested causal pathway: Exclusion increased social needs-threat, which in turn was associated with stronger disapproval of moral violations. When parsing by moral foundation, there was a significant indirect effect of social exclusion on harm, fairness, and sanctity violations but not for authority or loyalty violations. A possible reason for these differences between moral foundations is that the stimuli set from which we drew our moral vignettes found greater disapproval for harm, fairness, and sanctity violations, relative to authority and loyalty violations^19^. Because people judge violations of harm, fairness, and sanctity to be particularly reprehensible, these foundations may be more sensitive to threat. Nonetheless, the indirect effects of social threat on both authority and loyalty violations showed trends in the same direction as the other foundations.

## Study 2

While the indirect effect in Study 1 supported our hypothesis that social threat would amplify moral disapproval, this finding, along with the lack of a main effect, was unpredicted. For Study 2 we therefore conducted a direct replication and pre-registered our mediation model. Additionally, we included the MacArthur Ladder of Subjective Social Status^25^ to examine whether social status may act as a moderator between exclusion and fundamental needs. Prior research has found that higher-status individuals show reduced perceptual attunement to context and experience less uncertainty in social interactions, suggesting they may be less responsive to social threats^26^. We pre-registered two possibilities for the effect of social status: One was that the effect of exclusion would so powerful that it induces social threat in participants regardless of their subjective social status. Indeed, a meta-analysis of 120 studies showed an average effect size of d ≥ 1.40 for the Cyberball exclusion manipulation, suggesting that it is highly effective^4^. Another possibility is that individuals low in social status would be particularly responsive to exclusion and therefore needs-threat, and, as a result, more strongly condemn moral wrongdoers. Thus, the goal of Study 2 was to replicate the indirect effect obtained in Study 1 while also exploring the potential moderating effect of subjective social status.

### Methods

#### Participants

Because the experiment was a replication of Study 1 which showed an indirect effect of *b* = 0.26 across all moral items, we used a similar sample size of 432 for a power of 0.80. Participants were recruited using the same eligibility criteria as in Study 1. We collected data from 444 participants to account for possible exclusion, which was applied to 16 participants due to incorrect responses to both of the two manipulation check items. No other participants were excluded. Our final sample consisted of 428 participants (250 women; age: *M* = 34.03 years, *SD* = 11.53). The inclusion condition comprised 122 women and 92 men (age: *M* = 33.67 years, *SD* = 11.88) and the exclusion condition comprised 128 women and 86 men (age: *M* = 34.38 years, *SD* = 11.19). There were no significant differences between conditions for education, *t*(426) =  − 0.19, *p* = 0.852, income, *t*(426) = 1.03, *p* = 0.305, or political orientation *t*(426) =  − 0.06, *p* = 0.953.

#### Procedure

Identical materials, measures and procedure as in Study 1 were used, with the only additions being measures of education, income, and political orientation, along with the MacArthur Scale of Subjective Social Status^25^, administered after participants completed the Fundamental Needs Scale. Participants were shown an image of a ladder with 10 rungs, and instructed to think of the top of the ladder as consisting of individuals with prestigious occupations, high education, and high pay, while the bottom of the ladder represented individuals with less prestigious occupations, less education, and low pay. They then indicated their own current standing relative to others in the United States by selecting a rung. The pre-registration is here: http://aspredicted.org/blind.php?x=ay4js2. We report all measures, manipulations and exclusions. The pre-registration is here: http://aspredicted.org/blind.php?x=ay4js2. We report all measures, manipulations and exclusions.

### Results

#### Manipulation check

Socially included participants reported a higher percentage of received throws during the Cyberball game (*M* = 37%, 95% CI = [36%, 38%]) than those who were excluded (*M* = 13%, 95% CI = [13%, 14%]), *t*(426) =  − 32.83, p < 0.001. Additionally, they accurately reported more inclusion in the game (*M* = 4.92, 95% CI = [4.65, 5.18]), than excluded participants (*M* = 1.73, 95% CI = [1.51, 1.96]), *t*(426) =  − 17.86, p < 0.001.

#### Social needs-threat

The mediator variable was calculated as for Study 1. As expected, excluded participants reported lower social needs (*M* = 2.84, 95% CI = [2.68, 3.01]) than included participants (*M* = 5.21, 95% CI = [5.02, 5.41]), *t*(379) =  − 17.44, *p* < 0.001. Thus, as for Study 1, the manipulation was successful.

#### Moral judgment

Normality tests revealed that four sanctity items were substantially skewed^19^, which we had considered a possibility in the pre-registration, and they were therefore excluded from analyses. A two-way ANOVA with moral foundation (Harm, Fairness, Authority, Loyalty, and Sanctity) as within-subjects factor and condition (Inclusion vs. Exclusion) as a between-subjects factor showed a main effect of foundation, *F*(1, 426) = 49.80, *p* < 0.001, η^2^ = 0.11, with the highest ratings for sanctity (*M* = 3.55, 95% CI = [3.48, 3.62]) and fairness (*M* = 3.55, 95% CI = [3.49, 3.60]), followed by harm (*M* = 3.52, 95% CI = [3.46, 3.58]), authority (*M* = 3.41, 95% CI = [3.35, 3.48]), and loyalty violations (*M* = 3.20, 95% CI = [3.13, 3.27]). The relative ordering of severity of ratings across foundations therefore replicated the ordering obtained in Study 1. Also in line with the results of Study 1, there was no main effect of condition *F*(1, 426) = 2.19, *p* = 0.14, η^2^ = 0.00, nor any significant two-way interaction between foundation and condition, *F*(1, 426) = 0.316, *p* = 0.574. Thus, we again found that social exclusion as a variable on its own did not directly influence moral condemnation.

#### Needs-threat as a mediator

As in Study 1 and following our pre-registration, we performed a confirmatory analysis using bootstrapping^21^ to test the proposed mediation model that needs-threat acts as a mediator for the relationship between exclusion and moral disapproval. Cronbach’s alpha across the four needs was 0.92 (0.85 for belonging, 0.82 for self-esteem, 0.88 for meaningful existence, and 0.73 for sense of control), indicating a high level of internal consistency. As before, we included the composite needs-threat score as the mediating variable in the analysis.

**Figure 2:**
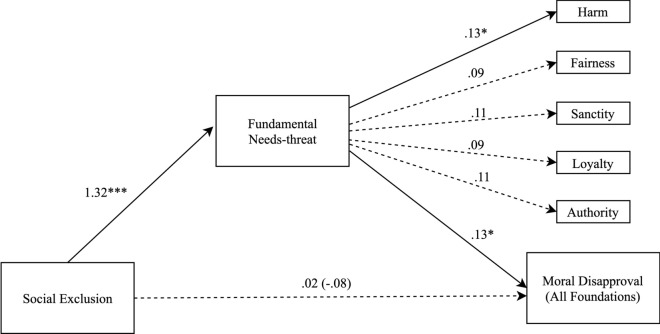
Indirect effect of social threat through fundamental needs-threat on moral disapproval for each moral foundation and across all moral violations in Study 2. Note *p < .05, **p < .01, ***p < .001. Solid lines are significant paths. Values are standardized path coefficients.

Replicating the key finding from Study 1 and in line with our preregistered hypothesis, 5,000 bootstrapped samples indicated that needs-threat mediated the relationship between exclusion and moral disapproval (*b* = 0.10, *SE* = 0.05; CI = 0.003 to 0.19), such that participants in the exclusion condition experienced increased needs-threat, and as a consequence, reported higher moral disapproval ratings (see Fig. 2 for standardized path coefficients, and Table 2 for direct and indirect effects).

**Table 2:** Study 2 Regression results for the proposed mediation of the effect of exclusion on moral disapproval by needs-threat.

Model	Estimate	SE	*p*	CI (lower)	CI (upper)
**Model without mediator**					
Intercept	3.40	.04	< .001	3.33	3.48
Exclusion → Moral Disapproval (*c*)	− .08	.05	.140	− .19	.03
R^2^_Y,X_	.01	–	–	–	–
**Model with mediator**					
Intercept	3.62	.11	< .001	3.40	3.83
Exclusion → Needs-threat (*a*)	2.37	.13	< .001	2.11	2.62
Needs-threat → Moral Disapproval (*b*)	.04	.02	.043	.001	.08
Exclusion → Moral Disapproval (*c*′)	.02	.07	.818	− .12	.16
Indirect effect (*a* X *b*)	.10	.05	–	.005	.19
Standardized indirect effect (*a* X *b*)	.17	.09	–	.009	.34
R^2^_M,X_	.44	–	–	–	–
R^2^_Y,MX_	.02	–	–	–	–

Regression weights a, b, c, and c′ are illustrated in Fig. 2. R^2^_Y,X_ is the proportion of variance in Y explained by X, R^2^_M,X_ is the proportion of variance in M explained by X, and R^2^_Y,MX_ is the proportion of variance in Y explained by X and M. The 95% CI for *a* X *b* is obtained by the bias-corrected bootstrap with 5,000 resamples. Exclusion is the independent variable (X), Needs-threat is the proposed mediator (M), and Moral Disapproval is the outcome (Y). CI (lower = lower bound of 95% confidence interval; CI (upper) = upper bound.

While we chose this mediation model based on theoretical reasoning that perceived social threat is a key variable in amplified moral judgments, other theoretical models might also be possible. Indeed, mediation analyses are consistent with, but do not prove, a hypothesized causal pathway of X → M → Y^27,28^. However, M (needs-threat) was measured after X (inclusion vs. exclusion), and it was the manipulation of X that then caused M as has been shown in a meta-analysis of 120 Cyberball studies^4^. Furthermore, M logically precedes Y (moral judgments): Needs-threat as a result of social exclusion occurred before the moral vignettes, which supports the hypothesized ordering^29^.

#### Moral foundations and needs-threat

Bootstrapping analyses revealed a significant indirect effect of exclusion on moral condemnation for harm (*b* = 0.11, *SE* = 0.05; 95% CI = 0.01 to 0.22) via needs-threat (see Fig. 2 for standardized path coefficients). Although the pattern was similar, effects were smaller and non-significant for fairness (*b* = 0.07, *SE* = 0.05; 95% *CI* =  − 0.03 to 0.18), sanctity (*b* = 0.11, *SE* = 0.07; 95% CI = -0.02 to 0.24), authority (*b* = 0.10, *SE* = 0.06; 95% CI = -0.01 to 0.21), and loyalty (*b* = 0.08, *SE* = 0.07; 95% CI =  − 0.04 to 0.21).

#### Subjective social status, exclusion, and needs-threat

We tested whether the indirect effect of exclusion on moral disapproval rating varied across levels of subjective social status, we used the PROCESS macro for moderated mediation analysis^21^. The model generates bias-corrected 95% bootstrap confidence intervals for the indirect effects using 5,000 bootstrap samples. Subjective social status did not moderate the relationship between exclusion and fundamental needs (*b* = 0.02, *SE* = 0.07, *p* = 0.748). Thus, the effect of exclusion on needs-threat did not vary with social status.

### Discussion

Confirmatory analyses in Study 2 replicated the results of the exploratory analyses of Study 1, namely the indirect effect of social needs-threat on moral condemnation when averaging across all moral items. Considering each moral foundation separately, the strongest effect occurred for harm violations, as in Study 1. This is consistent with the notion that increased social threat gives rise to stricter judgments of those who inflict harm because they may pose a greater threat relative to individuals who commit other types of moral transgressions. Importantly, subjective social status did not moderate the effect of social threat on fundamental needs.

## Study 3

To test whether the prospect of social threat, in addition to the experience of social threat, relates to moral condemnation, we conducted a further correlational study. Social anxiety constitutes the “anxiety resulting from the prospect or presence of personal evaluation in real or imagined social situations,” and can thus be considered a form of social threat^30^. Indeed, social anxiety results from both the possibility and the experience of social exclusion^31,32^, and is thought to motivate the preservation or enhancement of social acceptance amongst one’s peers and the avoidance of behavior that could result in being rebuffed^33,34^.

A commonly used scale to assess social anxiety is the Social Anxiety Questionnaire (SAQ) which assesses five dimensions: (1) Speaking in public/Talking with people in authority, (2) Interactions with attractive individuals or individuals of the opposite sex, (3) Assertive expressions of displeasure, (4) Criticism and embarrassment, and 5) Interactions with strangers^35^. The scale has been used for both clinical and non-clinical populations, with good internal consistency, construct validity, and cultural invariance^36^.

Based on results from Studies 1 and 2, for our third pre-registered study we hypothesized that individual differences in social anxiety would be associated with harsher moral judgments. In addition, we included the UCLA Loneliness Scale, widely used to measure the frequency of social interaction and emotions linked to loneliness^37^. We chose this scale to determine whether moral judgments are associated more with social *threat* (= social anxiety), or social *lack* (= loneliness) in an effort to add convergent validity to our hypothesis. Finally, we again included the MacArthur Scale of Subjective Social Status to test whether social status is associated with moral judgments, and to whether it acts as a moderator between social anxiety and moral judgment or loneliness and moral judgment.

### Methods

#### Participants

We recruited participants as in Studies 1 and 2. A G*Power analysis with 80% power at α = 0.05 to obtain an effect size of *r* = 0.30 suggested a sample size of 84. We collected data from 104 participants to account for the possibility of some participants providing unusable data. We excluded two participants for failing both of the two attention check items in one of the study questionnaires. Our final sample consisted of 102 participants (50 women; age: *M* = 33.20 years, *SD* = 10.35).

#### Social anxiety questionnaire

The SAQ is a 30-item instrument in which participants rate their unease, stress, or nervousness in different social situations on a scale from 1 (not at all) to 5 (extremely high). Sample items include “Greeting each person at a social meeting when I don’t know most of them” and “Telling someone I am attracted to that I would like to get to know them better.” It also contains two attention check items, including “Being mugged or robbed by an armed gang.” As recommended by the SAQ creators, we removed participants who selected “1” or “2” on both of the attention check items^35^.

#### UCLA loneliness scale

UCLA Loneliness Scale, Version 3 is a widely used 20-item loneliness assessment tool^37^. Sample questions include “How often do you feel that you are ‘in tune’ with the people around you?” and “How often do you feel alone?” Items were reverse-scored when necessary.

#### Moral judgment

We used the same vignettes as in Studies 1 and 2. Participants completed them first, and then received the two questionnaires in a counterbalanced order. The pre-registration is here: http://aspredicted.org/blind.php?x=8t4zj3, We report all measures, manipulations and exclusions. The pre-registration is here: http://aspredicted.org/blind.php?x=8t4zj3, We report all measures, manipulations and exclusions.

### Results

We excluded one sanctity item, as it was substantially skewed^20^, and then conducted correlational analyses between the scales. Social anxiety scores were normally distributed, with a skewness of 0.07 (*SE* = 0.24) and kurtosis of − 0.39 (*SE* = 0.47). Loneliness scores were normally distributed, with a skewness of − 0.07 (*SE* = 0.24) and kurtosis of 0.53 (*SE* = 0.47).

#### Social anxiety

**Figure 3:**
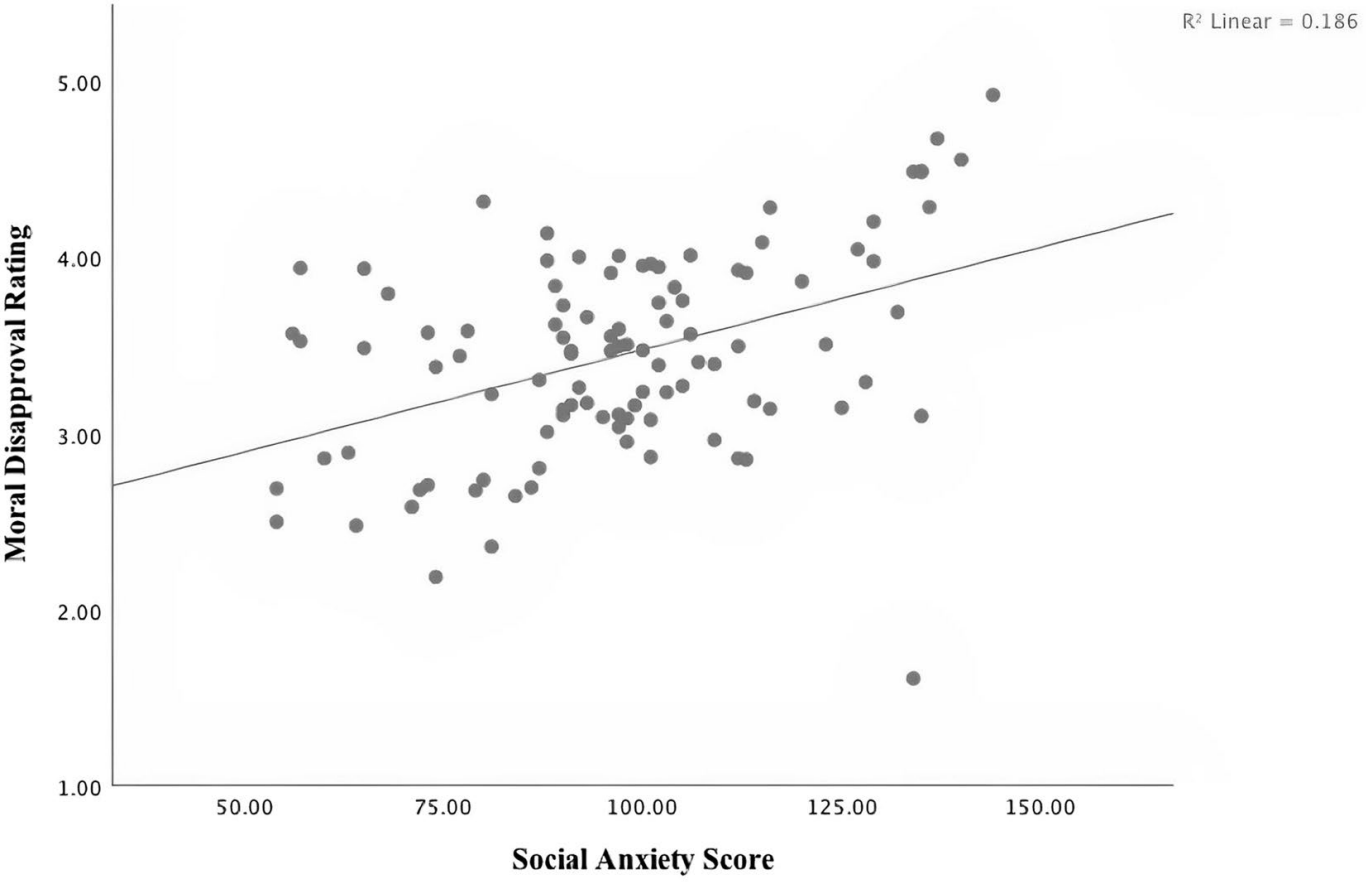
Scatterplot for Social Anxiety and Moral Disapproval for Study 3.

There was a significant correlation, *r*(102) = 0.43, *p* < 0.001, 95% CI = [0.26, 0.58]) between social anxiety and moral condemnation across all items (see Fig. 3 for scatterplot). Results were significant for harm (95% CI = [0.20, 0.53]), fairness (95% CI = [0.19, 0.53]), authority (95% CI = [0.24, 0.56]), loyalty (95% CI = [0.25, 0.57]), and sanctity (95% CI = [0.06, 0.42]). Thus, the association between social anxiety and moral condemnation held across all moral items, and for each individual moral foundation (see Table 3 for correlations between social anxiety and moral foundation).

**Table 3:** Correlations among variables for Study 3.

		1	2	3	4	5	6	7	8
1	Social Anxiety								
2	Loneliness	.20*							
3	All MFVs	.43***	− .01						
4	Harm	.38***	.01	.86***					
5	Fairness	.37***	− .09	.88***	.80***				
6	Authority	.41***	− .05	.90***	.67***	.74***			
7	Loyalty	.42***	.03	.84_***_	.57***	.60***	.81***		
8	Sanctity	.25*	.04	.78***	.64***	.62***	.59***	.53***	

*N* = 102. *p < .05; **p  ≤ .01; ***p  ≤ .001.

#### Loneliness

No significant correlation was observed for moral condemnation across all items, 95% CI = [−0.21, 0.18]), nor for any of the individual foundations, all *p*s > 0.372.

#### Social anxiety and loneliness

A hierarchical multiple regression was run to determine if the addition of loneliness improved the prediction of moral disapproval over and above social anxiety alone. There was no significant increase in R^2^, *F*(1, 99) = 1.288, *p* = 0.259. Thus, while social anxiety had a positive relationship with moral disapproval, including loneliness did not explain any further variance. Indeed, the correlation between the two variables was relatively low (*r* = 0.20), indicating that they indeed capture different underlying constructs.

#### Subjective social status

No significant correlation was observed for moral disapproval across all items, *r* = 0.06, *p* = 0.528, (95% CI = [−0.14, 0.27]), nor for any of the individual foundations, all *p*s > 0.131.

####  Social anxiety and subjective social status

Subjective social status was tested as a potential moderator between social anxiety and moral condemnation. Subjective social status did not moderate the relationship between social anxiety and moral condemnation (*b* = 0.00, *SE* = 0.00, *p* = 0.208). Thus, the association between social anxiety and moral condemnation did not vary with social status.

#### Subjective social status and loneliness

Similarly, subjective social status did not moderate the relationship between loneliness and moral condemnation (*b* = 0.00, *SE* = 0.00, *p* = 0.449.

### Discussion

As predicted in our pre-registration, social anxiety was correlated with moral disapproval at *r* = 0.43, considered to be a large effect size^38^. Furthermore, social anxiety had a similar association with all moral foundations violations. In contrast, we found no link between loneliness and moral disapproval ratings, implying that social threat, rather than social lack, is associated with harsher moral judgments. Furthermore, subjective social status was not correlated with moral judgment, nor did it moderate the associations between social anxiety and moral judgment or loneliness and moral judgment.

## General discussion

Taken together, findings from three pre-registered studies suggest that challenges to one’s social needs is associated with increased sensitivity to moral violations. Experimental evidence from Studies 1 and 2 suggests that exclusion activates social threat by thwarting fundamental social needs, which in turn was associated with harsher condemnation for moral wrongdoers. Separating by moral foundation, results revealed the most pronounced effect for harm violations, relative to fairness, authority, loyalty, and sanctity violations. Study 3 examined whether individual differences in social threat and loneliness would be associated with harsher moral judgments. While social anxiety was positively correlated with moral condemnation, loneliness was not, suggesting that social threat, rather than the absence of social connection, is associated with amplified moral condemnation.

These findings indicating that social threat is associated with harsher moral judgments suggest that various threats to survival can influence assessments of moral wrongdoing. Indeed, it has been proposed that the reason social exclusion reliably results in negative emotions is because social disconnectedness has been detrimental throughout human societies^6,7,9–11^. As we found in Studies 1 and 2 and consistent with prior research^4,5^, even brief exclusion via a simulated computer game can thwart fundamental social needs. Taken together, these experimental and correlational findings suggest that an elevated sense of danger appears to fortify moral judgment, because when safety is compromised, wrongdoers represent yet another source of potential danger. As a consequence, vulnerable individuals may be motivated to condemn moral violations more harshly. Interestingly, the null finding for loneliness suggests that amplified moral condemnation is not associated with having no social connections in the first place, but rather, with the existence or prospect of social threat. Relatedly, prior research has shown that greater cortisol release is associated with social anxiety but not with loneliness^7,39^ indicating that the body’s stress response does not react to loneliness in the same way as it does to social threat.

There are limitations within these findings. For example, though we replicated our results, there are possible factors that limit the benefits of using MTurk for data collection. MTurk is by far the most frequently used online data collection platform^40^ and contains a large and diverse participant pool^41^. Nevertheless, researchers have described its challenges, including inattention^42^, non-naïveté^43^, and vulnerability to “bots”^44^. To mitigate the possibility of low data quality, we adhered to recommended guidelines^45^ and recruited only MTurkers with an approval rating of at least 95%, and we included manipulation checks in Studies 1 and 2 and attention checks in Study 3.

Considering the implications of our results, we may speculate that the positive association between social threat and moral judgment is one reason why online moral outrage has recently intensified^46^. Expressing moral outrage can benefit society by holding wrongdoers accountable and broadcasting to others that such behavior is unacceptable. However, online settings might encourage a “piling on” effect, such that observers of a transgression may not consider proportionality when condemning the wrongdoer^47,48^. Social media facilitates frictionless reprobation, and as each additional individual contributes to public reprimand, the cumulative effect might be, depending on the transgression committed, greater than what the wrongdoer deserves. Along these lines, there is suggestive evidence that the desire the punish others makes them appear less human^49^. Social media might, by exacerbating moral outrage, intensify dehumanization of the target of disapproval. Lastly, expressing moral outrage may result in less involvement in social causes. Research on the moral licensing effect, for example, suggests that people implicitly monitor their moral value, and, once they have done something good, they later feel license to forego engaging in additional helpful behaviors^50,51^. Relatedly, when given the chance to express outrage with written messages, people are less likely to spend money to promote fair behavior^52^.

Internet-users often experience a relentless sense of social threat because social media content encourages upward social comparison, which might make them feel inferior to their peers^53^. At the same time, they often witness transgressions because social media is optimized to elicit moral outrage^54^. For example, people may feel socially unpopular when scrolling through social media profiles, and then, when learning about an infraction, express greater moral outrage than had they not experienced the erstwhile social threat. Thus, the combination of online activities of comparing oneself to others, along with reading news stories and social media posts designed to prompt moral outrage, may contribute to the current level of online vitriol.

On a more positive note, the current findings suggest that reducing the experience of social threat may be one way to curb excessive moral outrage. Indeed, prior research has found that moral judgment can be attenuated. Pharmacologically inhibiting disgust, for example, has been shown to reduce moral condemnation for sanctity violations^55^. Perhaps, in a similar manner, inhibiting social threat could reduce moral outrage. A possible approach could be to remind people of their supportive social relationships when they are experiencing social threat. For instance, earlier work has shown that reminders of positive social contacts moderate the perception of physical obstacles such that they appear less formidable, relative to reminders of neutral or negative social contacts^56^. Likewise, positive social contact may attenuate the effect of social threats. In short, if social threat exacerbates moral condemnation, bolstering social bonds could curb it.

## Supplementary Information

Supplementary Information.

## Data Availability

Preregistrations for all three studies can be accessed at http://aspredicted.org/blind.php?x=pu9xy2; http://aspredicted.org/blind.php?x=ay4js2, and http://aspredicted.org/blind.php?x=8t4zj3. All data are openly available on the following website https://osf.io/yjd37/?view_only=c62a729b4cb44dbf88c078b13a7b51af.
