# Supplementary material for: Social threat indirectly increases moral condemnation via thwarting fundamental social needs
Source: Sci Rep. 2021 Nov 5;11:21709. doi: 10.1038/s41598-021-00752-2 (PMC8571390; doi:10.1038/s41598-021-00752-2)
Supplement: Supplementary file 1 — Supplementary Information. [file 41598_2021_752_MOESM1_ESM.docx]

**Supplementary Information**

**Pilot Study**

The same Cyberball procedure followed by moral evaluations as in Studies 1 and 2 was used, with the exception that we only included items from the Harm and Fairness foundations (12 items each) from the Clifford et al. (2015) stimulus set. Despite constituting standardized vignettes normed to show responses in the mid-range of the scale, however, nine out of the 24 vignettes unexpectedly contained an excessive number of extreme responses, leading to highly negatively skewed distributions. Excluding these items reduced the available data substantially, and we therefore did not consider the results of the study to be conclusive. Nevertheless, we report the method and results below because we observed a suggestive effect for the Harm foundation and used its magnitude to calculate the sample sizes reported in the main text.

***Method***

***Participants*.** Participants were recruited on the online testing platform Prolific. We aimed to test approximately 100 participants for each of the two conditions (200 total). Data were collected from 220 participants to account for the possibility of some not following instructions. We excluded two for failing to complete the online consent form prior to the start of the task. The final sample consisted of 218 participants (154 women, 64 men; age: *M* = 35.57 years, *SD* = 12.11).

***Procedure****.* The same procedure as in Studies 1 and 2 was used. As an exploratory measure, participants completed the Ten Item Personality Inventory (Gosling, Rentfrow, & Swann, 2003).

***Results***

***Manipulation Check*.** Participants accurately estimated the percentage of throws they received, with higher percentages of received throws for included participants (*M* = 40%, 95% CI = [38%, 43%]) than excluded participants (*M* = 15%, 95% CI = [13%, 16%]), *t*(216) = -19.89, p < .001. Additionally, the former accurately perceived higher inclusion in the game (*M* = 4.98, 95% CI = [4.61, 5.36]), than the latter (*M* = 1.79, 95% CI = [1.46, 2.13]), *t*(216) = -12.60, p < .001. Thus, the manipulation was effective.

***Needs-Threat*.** A composite score (Cronbach’s alpha of .92) was formed as in the main studies, and subscales assessing each need were examined, indicating a high level of internal consistency with Cronbach’s alpha of.88 for belonging, .86 for self-esteem, .88 for meaningful existence, and .72 for sense of control. As expected, excluded participants reported lower fundamental needs (M = 2.81, 95% confidence interval, or CI = [2.58, 3.03]) than included participants (M = 4.92, 95% CI = [4.68, 5.16]), *t*(216) = -12.73, p < .001.

***Moral judgment*.** We unexpectedly found score limitations for moral violations such that participants rated many items to be a 4 (“very wrong”) or 5 (“extremely wrong”), resulting in negatively skewed distributions. This limitation was unanticipated because we used items that were validated and recommended for general use (Clifford et al., 2015). Tests of normality revealed that nine out of the 24 vignettes contained skewness values lower than -1, reflecting substantially skewed distributions (Hair et al., 2013). We therefore excluded these items from the analyses, which led to an unequal number of items for the two foundations (6 for Harm, 9 for Fairness). With this caveat in mind we proceeded to analyze the remaining data to test whether there was any suggestive evidence for our hypothesis.

In a two-way ANOVA with moral foundations vignettes (Harm and Fairness) as a within-subjects factor and condition (included or excluded) as a between-subjects factor, mean disapproval rating varied significantly as a function of moral foundation, F(1, 216) = 7.25, p = .008, η^2^ = 0.03, but there was no effect for condition, F(1, 216) = 2.68, p = .103, η^2^ = 0.12, nor any two-way interaction between vignette type and condition, F(1, 216) = 0.514, p = .474. However, an analysis comparing the exclusion versus inclusion conditions specifically for harm violations revealed a non-significant trend of an effect, with *d* = 0.24, *p* = .086.

***Discussion***

The pilot study did not reveal a statistically significant main effect of social threat on moral judgments, but suggested a possible influence specifically for Harm violations. However, we considered the results inconclusive because there were unexpected ceiling effects for nine out of 24 moral vignettes. Furthermore, we had used only two moral foundations and we had used a relatively small sample size, which might not have provided enough statistical power to reliably detect the observed small effect size. To address these limitations, we designed Study 1 to reflect the procedural dynamics of the pilot study, but replaced items that were at ceiling with less severe moral violations, and added vignettes from the loyalty, authority, and sanctity moral foundations. For the main studies we also increased the sample size to account for the possibility of a small effect.

**Study 1**

***Exploratory Moderation Analysis***

As noted in the pre-registration, we included the Ten Item Personality Inventory (Gosling, Rentfrow, & Swann, 2003) as an exploratory measure to investigate whether any of the Big Five personality traits might moderate the relationship between social exclusion and moral judgment. Table 1 shows the unstandardized coefficients (b) and standard errors (SE) of the variables (independent variable – exclusion; moderator – personality trait; and interaction – exclusion X personality trait) of the moderation model, which revealed no significant interactions between social exclusion and personality traits on moral judgments.

**Table 1. Moderation analysis (*N* = 381)**

|  | | ***b*** | | | | | **SE** | | | ***t*** | | ***p*** | | |  |
| --- | --- | --- | --- | --- | --- | --- | --- | --- | --- | --- | --- | --- | --- | --- | --- |
| Constant (Openness) | | | 3.29 | | | .10 | | 33.21 | | | <.001 | | |  |  |
| Exclusion | | | .01 | .06 | | | | .12 | | | .902 | | |  |  |
| Openness | | | -.11 | .08 | | | | -1.32 | | | .187 | | |  |  |
| Exclusion X Openness | .06 | | | | .05 | | | | 1.26 | | | | .208 | |  |
| Constant (Conscientiousness) | | | 3.28 | .10 | | | | 33.93 | | | <.001 | | |  |  |
| Exclusion | | | .01 | .06 | | | | .22 | | | .826 | | |  |  |
| Conscientiousness | | | -.24 | .08 | | | | -3.06 | | | .002 | | |  |  |
| Exclusion X Conscientiousness | | | .09 | .05 | | | | 1.85 | | | .065 | | |  |  |
| Constant (Extraversion) | | | 3.29 | .10 | | | | 33.93 | | | <.001 | | |  |  |
| Exclusion | | | .01 | .06 | | | | 0.13 | | | .894 | | |  | |
| Extraversion | | | .09 | .06 | | | | 1.58 | | | .116 | | |  | |
| Exclusion X Extraversion | | | -.07 | .04 | | | | -1.97 | | | .05 | | |  | |
| Constant (Agreeableness) | | | 3.29 | .10 | | | | 33.70 | | | <.001 | | |  | |
| Exclusion | | | .01 | .06 | | | | .18 | | | .860 | | |  | |
| Agreeableness | | | -.24 | .09 | | | | -2.75 | | | .006 | | |  | |
| Exclusion X Agreeableness | | | .10 | .05 | | | | 1.84 | | | .067 | | |  | |
| Constant (Emotional Stability) | | | 3.28 | .10 | | | | 32.95 | | | <.001 | | |  | |
| Exclusion | | | .01 | .06 | | | | .18 | | | .860 | | |  | |
| Emotional Stability | | | -.10 | .07 | | | | -1.23 | | | .219 | | |  | |
| Exclusion X Emotional Stability | | | .05 | .04 | | | | 1.19 | | | .234 | | |  | |

Note: No significant interactions, all *p*s > .05

**Reference**

Gosling, S. D., Rentfrow, P. J., & Swann Jr, W. B. (2003). A very brief measure of the Big-Five personality domains. *Journal of Research in Personality*, *37*, 504–528.
